# Microenvironmental effects limit efficacy of thymoquinone treatment in a mouse model of ovarian cancer

**DOI:** 10.1186/s12943-015-0463-5

**Published:** 2015-11-09

**Authors:** Andrew J. Wilson, Jeanette Saskowski, Whitney Barham, Dineo Khabele, Fiona Yull

**Affiliations:** Department of Obstetrics and Gynecology, Division of Gynecologic Oncology, Vanderbilt University School of Medicine, Nashville, TN USA; Department of Cancer Biology, Vanderbilt University Medical Center, Nashville, TN USA; Vanderbilt-Ingram Cancer Center, Vanderbilt University Medical Center, Nashville, TN USA; Department of Obstetrics and Gynecology, Vanderbilt University Medical Center, B1100 Medical Center North, Nashville, TN 37232 USA

**Keywords:** NF-κB activity, Ovarian cancer, Syngeneic mouse model, Macrophages, Thymoquinone, VEGF

## Abstract

**Background:**

Ovarian cancer is the most lethal gynecologic malignancy, with limited treatment options for chemoresistant disease. An important link between inflammation and peritoneal spread of ovarian cancer is NF-κB signaling. Thymoquinone (TQ) exerts multiple anti-tumorigenic cellular effects, including NF-κB inhibition. We aimed to investigate the therapeutic potential of TQ in an established murine syngeneic model of ovarian cancer.

**Methods:**

ID8-NGL mouse ovarian cancer cells stably expressing an NF-κB reporter transgene were injected intra-peritoneally into C57BL/6 mice, and mice were treated with TQ or vehicle for 10 or 30 days. TQ was combined with the macrophage depleting drug, liposomal clodronate, in selected experiments. Effects on peritoneal tumor burden were measured by volume of ascites, number of peritoneal implants and mesenteric tumor mass. NF-κB reporter activity and markers of proliferation and apoptosis were measured in tumors and in confirmatory *in vitro* experiments. Protein or mRNA expression of M1 (anti-tumor) and M2 (pro-tumor) macrophage markers, and soluble cytokine profiles, were examined from harvested ascites fluid, peritoneal lavages and/or tumor sections. 2-tailed Mann–Whitney tests were used for measuring differences between groups in *in vivo* experiments.

**Results:**

Consistent with its effects *in vitro*, TQ reduced proliferation and increased apoptosis in ID8-NGL tumors after 10 and 30 day treatment. Prolonged TQ treatment did not significantly alter tumor number or mass compared to vehicle, but rather exerted an overall deleterious effect by stimulating ascites formation. Increased ascites was accompanied by elevated NF-κB activity in tumors and macrophages, increased pro-tumor M2 macrophages and expression of pro-tumorigenic soluble factors such as VEGF in ascites fluid, and increased tumor infiltration of M2 macrophages. In contrast, a 10 day exposure to TQ produced no ascites, and reduced tumor NF-κB activity, M2 macrophages and soluble VEGF levels. Peritoneal macrophage depletion by clodronate significantly reduced tumor burden. However, TQ-stimulated ascites was further enhanced by co-treatment with clodronate, with macrophages present overwhelmingly of the M2 phenotype.

**Conclusions:**

Our findings show that pro-tumorigenic microenvironmental effects limited the efficacy of TQ in a syngeneic mouse model of ovarian cancer, and provide caution regarding its potential use in clinical trials in ovarian cancer patients.

**Electronic supplementary material:**

The online version of this article (doi:10.1186/s12943-015-0463-5) contains supplementary material, which is available to authorized users.

## Background

Ovarian cancer is the most common cause of death from gynecologic malignancies in the United States [1]. Most women with epithelial ovarian cancers are diagnosed with advanced, metastatic disease characterized by widespread peritoneal carcinomatosis and abdominal ascites [2]. The development of chemoresistance is common in advanced disease [3]. Therefore, identifying new drug treatment strategies is critical to prolonging the life of women with refractory disease.

The nuclear factor-kappaB (NF-κB) signaling pathway plays an important role in progression of multiple solid malignancies, including ovarian cancer. Constitutive activation of NF-κB is observed in a large subset of ovarian tumors, is associated with tumor growth, progression and resistance to chemotherapy, and is an important molecular link between inflammation and cancer [4–9]. In a syngeneic model of ovarian cancer using mouse ID8 cells stably expressing the NGL NF-κB reporter plasmid, we recently showed that NF-κB activity markedly increases during abdominal cancer spread and is reduced by treatment with the promising anti-cancer drug, thymoquinone (TQ) [10]. TQ, derived from the medicinal plant *Nigella sativa*, is a known inhibitor of NF-κB [11–14] which induces co-operative anti-tumor effects with the chemotherapeutic drug cisplatin in our ID8-NGL model [15]. Early clinical trials have shown promising lack of toxic effects in patients with cardiovascular disease [13], and in cancer patients [16]. Definitive trials for establishing safe and effective doses of TQ in cancer patients are currently lacking, but are well supported by preclinical data [11–14].

Equally relevant to cancer therapy, but less understood, are the possible effects of systemic NF-κB inhibition in the non-tumor cells of the host. Ovarian tumors are known to polarize macrophages in the tumor microenvironment to display pro-tumorigenic characteristics via aberrant NF-κB signaling activity [17, 18]. Classically activated or cytotoxic anti-tumorigenic macrophages (also called M1) and “alternatively” activated pro-tumorigenic macrophages (M2) represent two extremes in the spectrum of the macrophage phenotype [19]. This polarization is part of a complex interplay of signaling and responses between tumor cells and inflammatory cells such as macrophages, T cells and dendritic cells [20–22]. Targeting M2-like, tumor-associated macrophages for “re-education” towards a cytotoxic (M1), anti-tumor function by NF-κB inhibition is a promising therapeutic strategy [18]. Supporting this, we have recently shown that brief TQ exposure induces a shift towards M1 markers in peritoneal macrophages harvested from ascites fluid [10]. However, major gaps in knowledge still remain regarding the specific influence of NF-κB, and the consequences of inhibiting its activity, in cancer cells and host cells during tumorigenesis. This study aims to investigate the therapeutic potential of TQ in ovarian cancer progression in the ID8-NGL syngeneic model.

Our results show that TQ induced direct anti-tumor effects in ovarian cancer cells grown *in vitro*, and as syngeneic tumors in mice. However, the overall effect of prolonged TQ treatment was deleterious *in vivo*, characterized by rapid accumulation of ascites. Increased ascites was accompanied by a paradoxical increase in NF-κB activity in tumors and macrophages, increased M2 macrophages, elevated expression of pro-tumorigenic soluble factors such as VEGF, IL-10 and MCP-1 in ascites fluid, and increased infiltration of pro-tumor M2 macrophages into tumors. When peritoneal macrophages were depleted by liposomal clodronate, the ability of TQ to stimulate ascites formation was further enhanced, with the macrophages present overwhelmingly of the M2 phenotype. These findings show that pro-tumorigenic microenvironmental effects limited the efficacy of TQ, which provide strong caution regarding its use in future clinical trials in ovarian cancer patients.

## Results

### Anti-tumor effects of NF-κB inhibitor thymoquinone (TQ) in ID8-NGL cells

**Fig. 1:**
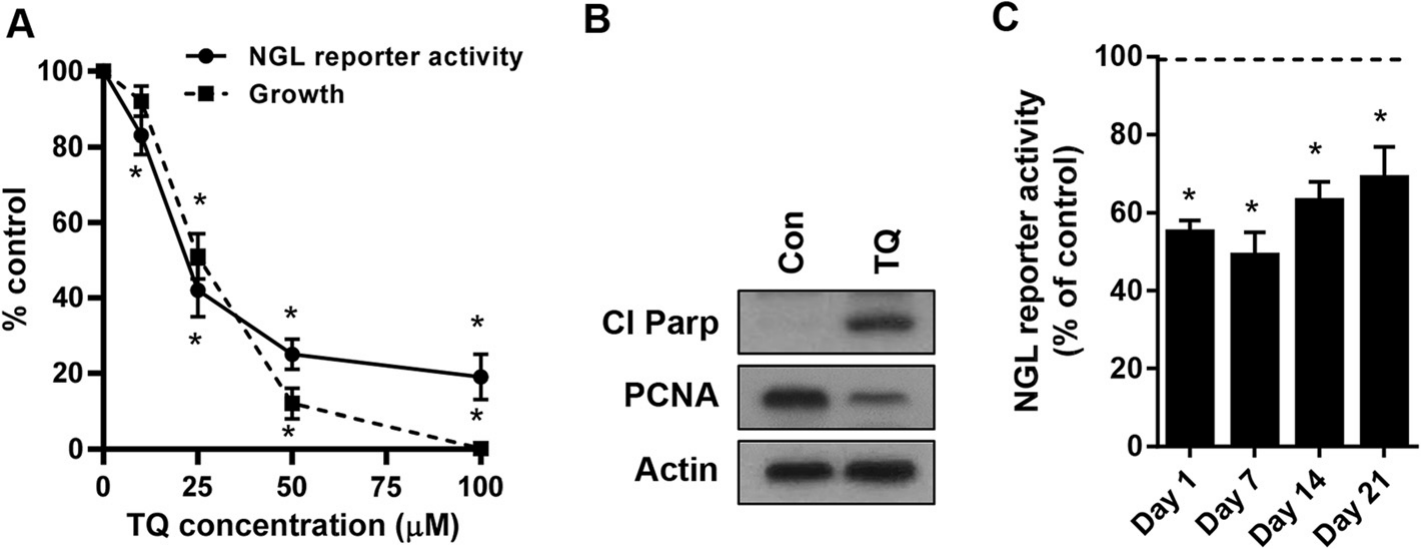
TQ reduces NF-κB activity and cell growth, and induces apoptosis in cultured ID8-NGL cells. **a** Effects of increasing concentrations of TQ on luciferase activity of the NGL reporter (24 h) and on cell growth measured in SRB assays (72 h), expressed as a percentage of control; *p < 0.02 compared to vehicle, Student’s *t* test. **b** Levels of apoptosis and proliferation in cells treated with 50 μM TQ (24 h) measured by western blot analysis of cleaved PARP and PCNA expression, respectively. Actin was used as a loading control. **c** Luciferase activity of the NF-κB reporter measured in ID8-NGL cells cultured *in vitro* treated with TQ 50 μM for the indicated time periods. Values are mean + SE. *p < 0.01 relative to control; Student’s test

We have previously shown that NF-κB activity in tumor cells increases during ovarian cancer progression using mouse ovarian cancer cells stably expressing an NF-κB-dependent GFP/luciferase reporter, ID8-NGL [10]. The promising anti-cancer drug, TQ, is a known inhibitor of NF-κB which synergizes with the chemotherapeutic drug cisplatin in ID8-NGL cells [15]. Here, we show that TQ inhibited ID8-NGL cell growth and viability in SRB assays, and NF-κB activity in luciferase assays, in a concentration-dependent manner (Fig. 1a). Moreover, the anti-tumor effects of TQ were associated with reduced cell proliferation and increased apoptosis by western blot analysis of PCNA and cleaved PARP, respectively (Fig. 1b).

We recently reported the ability of TQ to synergize with cisplatin and limit tumor progression [15]. However, TQ treatment also leads to an unexpected increase in ascites accumulation. In order to understand the mechanisms leading to this undesirable clinical outcome, we performed experiments examining effects of 10 day and 30 day treatment in parallel, starting 30 days after ID8-NGL cell injection.

**Fig. 2:**
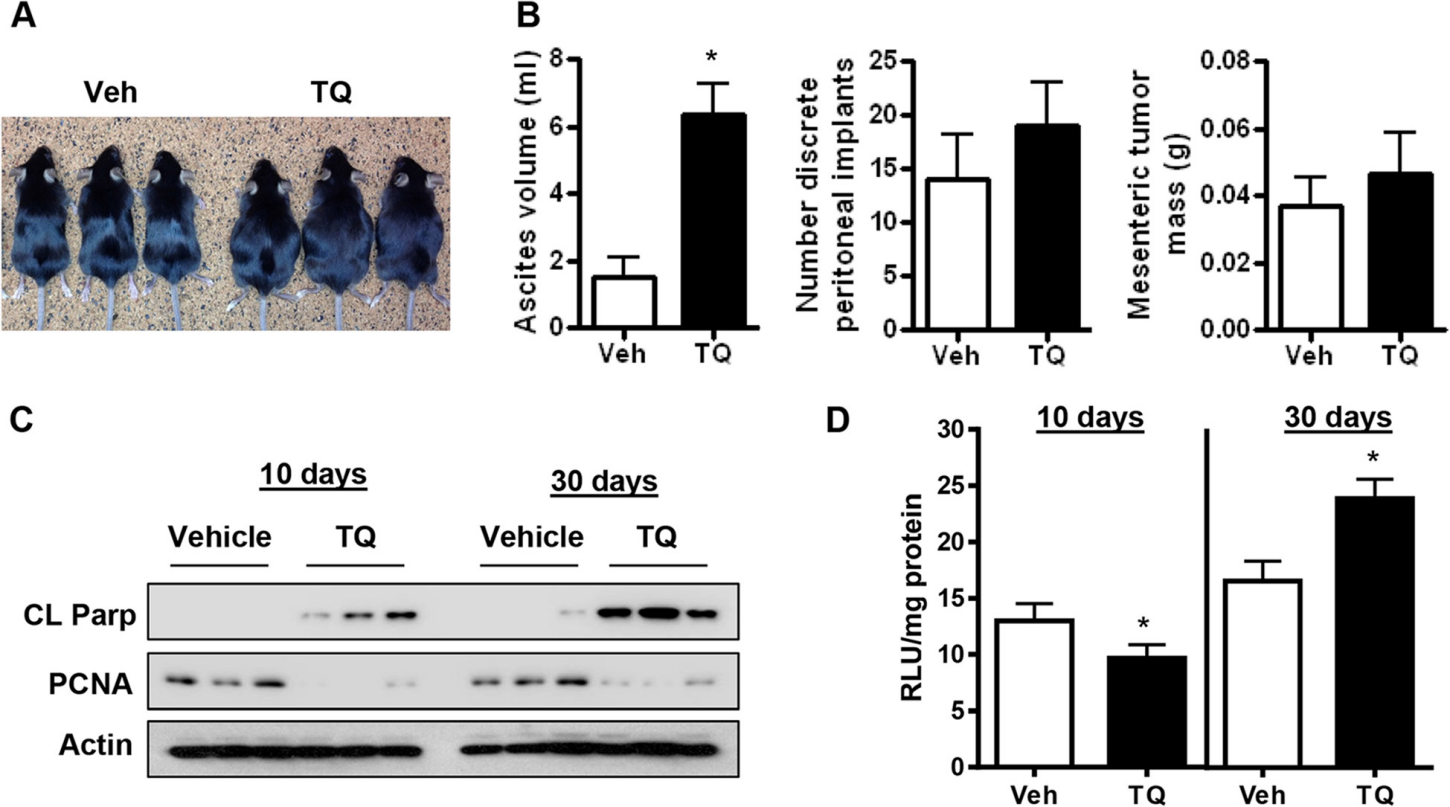
Effects of short- and long-term TQ treatment in ID8-NGL tumors. **a** Representative vehicle or TQ-treated mice after 30 days treatment. **b** Volume of ascites, number of peritoneal implants and mesenteric tumor mass in vehicle or TQ-treated mice after 30 days. **c** Protein levels of the apoptotic marker, cleaved PARP (CL PARP) and proliferation marker, PCNA, and **d** luciferase activity of the NGL reporter, in tumors harvested from mice treated with thrice weekly 20 mg/kg TQ or vehicle (Veh) for 10 or 30 days. *p < 0.01 compared to vehicle; Mann–Whitney test

Indices of tumor burden were not quantifiable due to insufficient tumor load after only 10 days treatment with vehicle or TQ. While 30 day TQ treatment had no effect on the overall number of peritoneal implants and mesenteric tumor mass, it did lead to increased levels of ascites (Fig. 2a and b). This observation was confirmed in a complementary model where wild-type ID8 cells were grown in BL/6 NGL reporter mice treated with TQ for 30 days (Additional file 1: Figure S1A-C). Increased ascites with TQ treatment was unlikely to be due to systemic toxic effects, since there were no overt signs of drug toxicity in behavior or body condition score of the whole animal, or on gross or histological examination of various organs at sacrifice (data not shown). We compared effects of short and long-term TQ treatment on cell proliferation and apoptosis in ID8-NGL tumors. At both time points, we observed reduction in expression of the proliferation marker PCNA, and increased expression of cleaved PARP (Fig. 2c), consistent with our *in vitro* data (Fig. 1b).

### Effects of TQ on NF-κB activity in ID8-NGL tumors

**Fig. 3:**
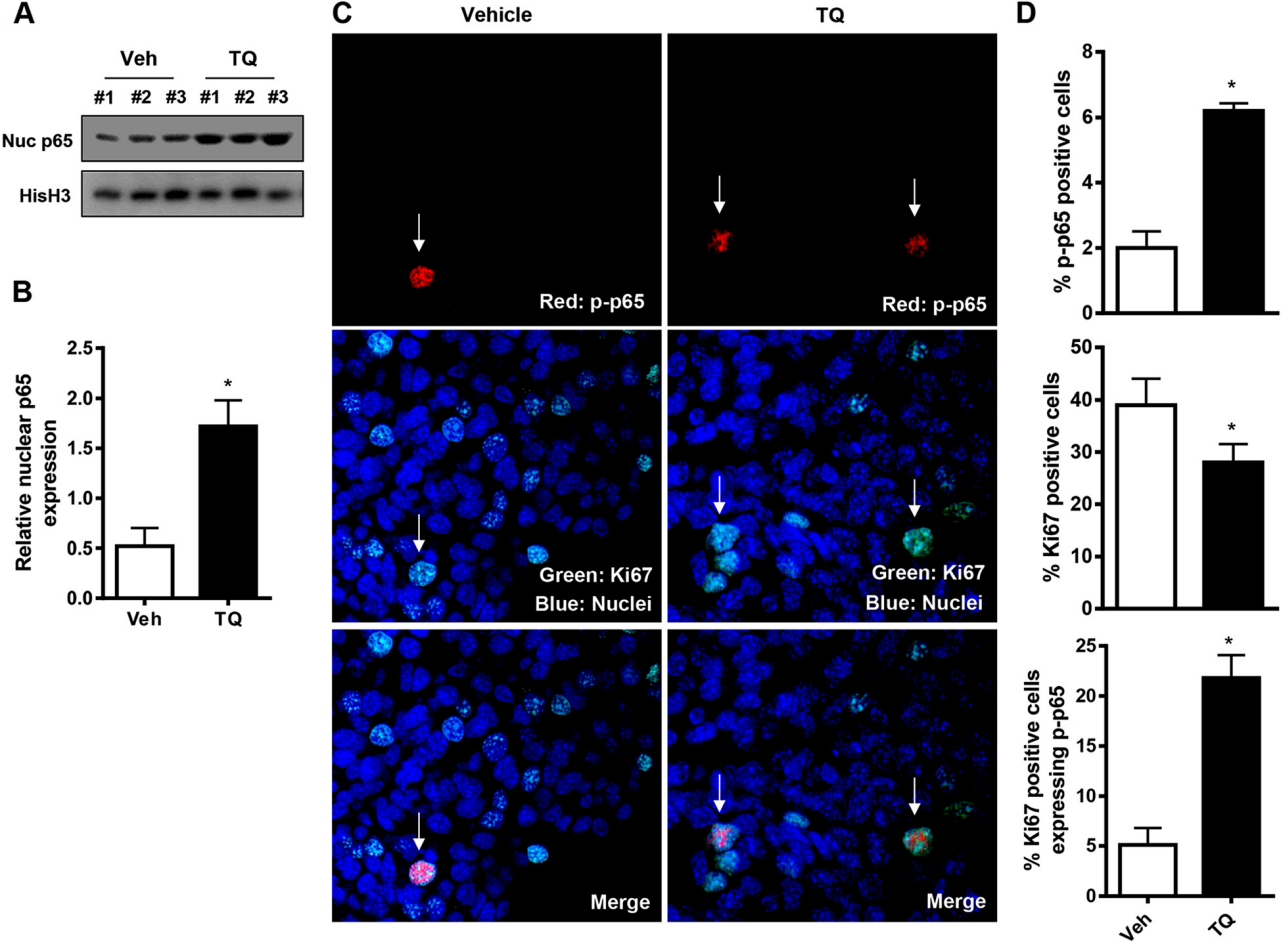
Effects of long-term TQ treatment on NF-κB activity in tumors. **a** Western blot analysis of p65 in nuclear extracts from ID8-NGL tumors harvested from mice treated with thrice weekly 20 mg/kg TQ or vehicle (Veh) for 30 days. Equal loading was shown by probing for the nuclear-specific protein, histone H3. **b** Nuclear p65 expression relative to corresponding histone H3 levels was measured by densitometry. **c** Immunofluorescent detection of the proliferation marker Ki67/mib-1 and the phosphorylated p65 (p-p65). DAPI-stained nuclei are in blue. Co-expression of phospho-p65 (p-p65; red) and Ki67/mib-1 (green) is observed in the nuclei of a subset of cells. **d** The percentage of tumor cells positive for Ki67 staining or nuclear p-p65, and the percentage of Ki67-positive cells also expressing nuclear p-p65. At least 200 cells were counted in 5 independent fields (×40 objective) for each drug treatment per mouse. Values are mean + SD for 3 mice per group *p < 0.01 relative to vehicle-treated mice, Mann–Whitney test

We observed contrasting, time-dependent effects of TQ on NF-κB reporter activity in ID8-NGL tumors in luciferase assays. As expected, NF-κB reporter activity was reduced with 10 day treatment (Fig. 2d), but prolonged treatment (30 days) resulted in a major change in effects, with increased NF-κB activity detected in tumors by both luciferase assay (Fig. 2d) and expression of p65 in nuclear fractions (Fig. 3a and b). Since upregulation of NF-κB activity is an established mechanism of drug resistance [23], we speculated that this may have limited the tumor response to TQ. To that end, we dissected patterns of NF-κB activity in tumor sections at a single cell level in immunofluorescence assays measuring expression of phosphorylated nuclear p65 (p-p65), an established marker of NF-κB activation. As shown in Fig. 3c and d (top panel), TQ significantly increased the percentage of cells staining positive for nuclear p-p65. Overall, TQ reduced the percentage of tumor cells expressing the proliferation marker Ki67 (Fig. 3c and d, middle panel), consistent with its effects on PCNA expression in our western blot analysis (Fig. 2c). Strikingly, however, the percentage of proliferating cells also displaying p-p65 expression was markedly increased by TQ (Fig. 3c and d, bottom panel). These data suggest that there was a subset of tumor cells with active NF-κB signaling that was no longer effectively being inhibited by the TQ treatment, i.e. a drug-resistant subpopulation.

To rule out the possibility that the contradictory effects of long-term TQ treatment were due to loss of potency of our TQ stock, we treated cultured ID8-NGL cells with thrice weekly TQ from the same stock for up to 21 days. As shown in Fig. 1c, there was persistence of TQ-inhibition of NGL reporter activity over this time period. This result also suggests that elevation of tumor NF-κB activity in 30 day TQ-treated mice was not a direct effect of the drug on the tumors.

### Long-term TQ treatment induces an increase in M2 macrophage markers in ascites fluid

Our observations that prolonged exposure of ID8-NGL cells to TQ *in vitro* was associated with persistent NF-κB inhibition (Fig. 1c), and the similar effects of 10 and 30 day TQ treatment on tumor proliferation and apoptosis (Fig. 2c), strongly suggest that direct tumor effects could not explain the deleterious effects of TQ, namely increased ascites formation associated with increased tumor NF-κB activity. We therefore speculated that TQ effects on the tumor microenvironment, secondary to direct tumor effects, may underlie these observations.

**Fig. 4:**
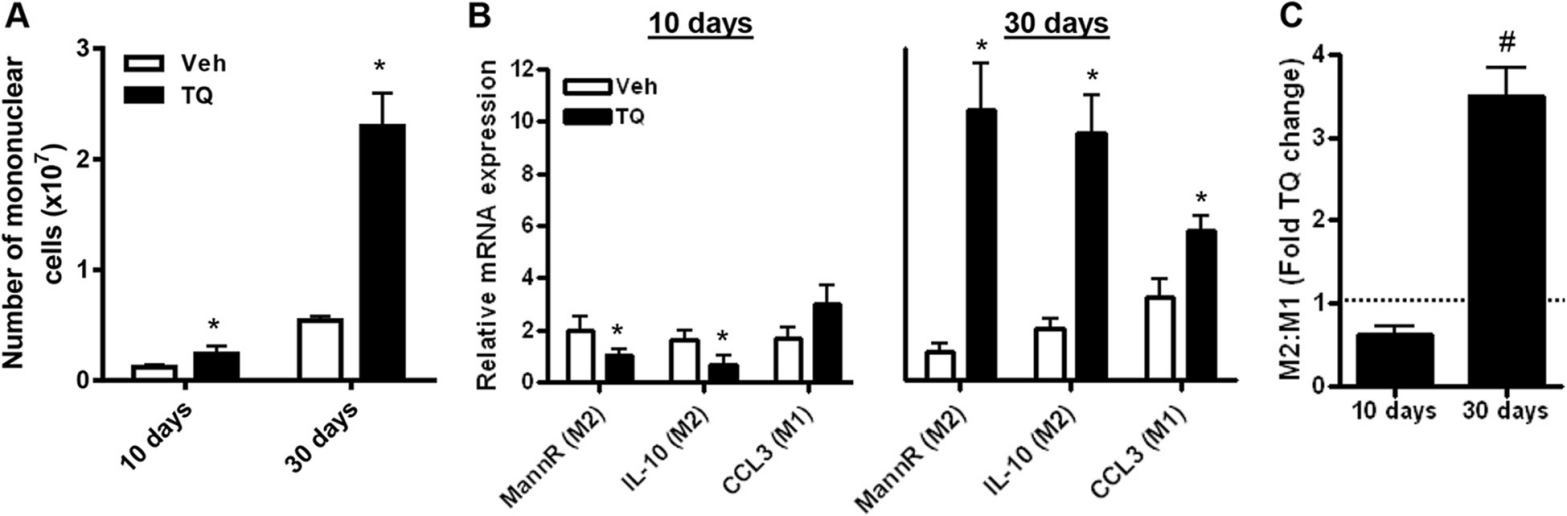
Effects of short- and long-term TQ treatment in macrophages isolated from ascites fluid. **a** Overall number of mononuclear cells increased in ascites collected from mice treated with thrice weekly 20 mg/kg TQ or vehicle (Veh) for 10 or 30 days, as quantified in differential cell counts of H&E-stained cytospin slides. **b** QPCR measurement of mRNA levels of established M2 (mannose-receptor and IL-10) and M1 (CCL3) macrophage markers. Values were expressed relative to corresponding GAPDH levels. **c** Ratio of M2 (mannose-receptor and IL-10) to M1 (CCL3) markers expressed as fold TQ-induced change in harvested peritoneal macrophages. *p < 0.01 compared to vehicle; #*p* < 0.01 compared to all other tumor types; *p < 0.01, both Mann–Whitney test

**Fig. 5:**
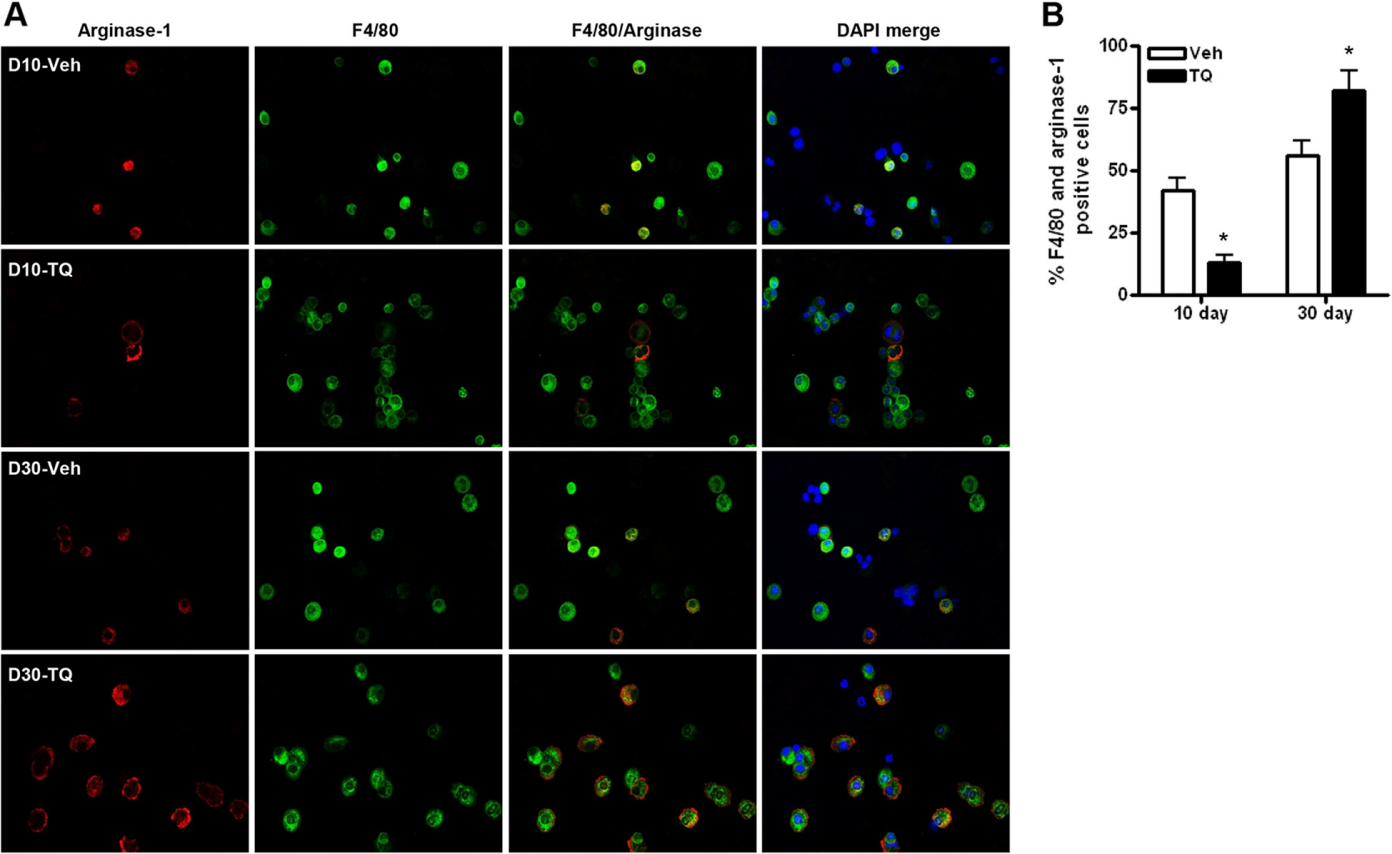
Effects of short- and long-term TQ treatment on arginase-1 expression in macrophages. **a** IF analysis of single cell suspensions of isolated macrophages from vehicle and TQ-treated mice. Expression of the macrophage markers F4/80 (green) and arginase-1 (red) was assayed. DAPI-stained nuclei are in blue. **b** Quantification of the percentage of cells staining positive for F4/80 and arginase-1. Values were determined from 5 representative fields at high power (×40). *p < 0.01 relative to vehicle; all *p* values determined by Mann–Whitney test

We have previously shown that mononuclear cells, particularly macrophages, are the predominant inflammatory cell population in the peritoneal cavity of ID8-NGL tumor-bearing mice [10]. Furthermore, M2-like macrophages are known to be “programmed” through signals from the tumor and other inflammatory cells to induce pro-tumor effects [19]. Therefore, we first examined TQ effects on macrophage number and phenotype in ascites fluid in our ID8-NGL tumor model. Morphological analysis of cytospin slides demonstrated an overall increase in mononuclear cells with TQ treatment at both time points, although the relative stimulatory effect was greater at 30 days (Fig. 4a), consistent with an elevated inflammatory response. QPCR analysis of macrophages isolated from ascites fluid showed that 10 day TQ treatment significantly reduced mRNA expression of established markers of pro-tumorigenic M2-like macrophages (mannose-receptor and IL-10). In contrast, 30 day TQ treatment markedly increased expression of these M2 markers, but also modestly increased mRNA expression of an anti-tumorigenic M1-like marker, CCL3 (Fig. 4b). Overall, however, there was a clear shift towards an M2 macrophage phenotype with 30 day TQ treatment (Fig. 4c). This result was confirmed at the protein level, where expression of the M2 marker, arginase-1, in macrophages was assessed by immunofluorescence. In cells staining positive for the established macrophage marker, F4/80, 30 day TQ treatment significantly increased the population of cells showing cytoplasmic staining for arginase-1, in contrast to 10 day treatment (Fig. 5a and b). A similar pattern of up-regulation of M2 macrophage markers was also observed in isolated macrophages from ID8-injected NGL reporter mice treated with TQ for 30 days (Additional file 1: Figure S1D).

### Analysis of macrophage infiltration into tumors

**Fig. 6:**
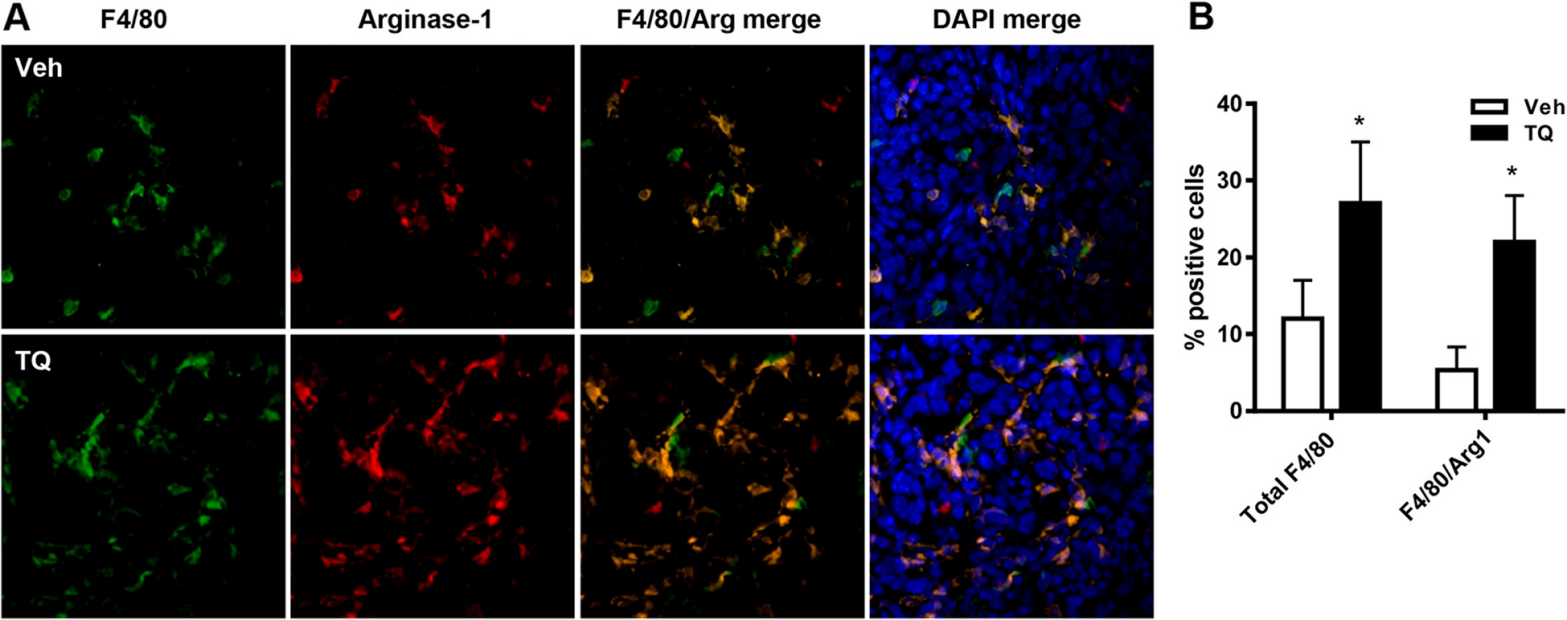
Macrophage infiltration in tumors in TQ-treated mice. BL/6 mice were injected with ID8-NGL cells and treated with vehicle, TQ (20 mg/kg), cisplatin (2 mg/kg) or the combination. **a** IF analysis of expression of the macrophage markers F4/80 (green) and arginase-1 (red) in paraffin-embedded tumor sections. DAPI-stained nuclei are in blue. **b** Quantification of the percentage of total cells positive for F4/80 and cells co-staining for F4/80 and arginase-1. Values were determined from 5 representative fields at high power (×40). Values are mean + SE for 3–5 mice per group. *p < 0.01 relative to vehicle; all *p* values determined by Mann–Whitney test

We have previously demonstrated extensive macrophage infiltration into intraperitoneal tumors derived from ID8-NGL cells [10]. In order to assess whether TQ effects on tumor burden were reflected in changes in macrophage infiltration and/or macrophage populations within the tumor, we measured expression of the well-established macrophage marker, F4/80, and the M2 macrophage marker, arginase-1, in formalin-fixed tumor sections. As shown in Fig. 6a and b, 30 day TQ treatment significantly increased the overall macrophage infiltration, measured by the percentage of F4/80-positive cells, and the percentage of F4/80-positive cells co-staining positive for cytoplasmic arginase-1 expression.

### Macrophage depletion by clodronate augments TQ-stimulated ascites formation

**Fig. 7:**
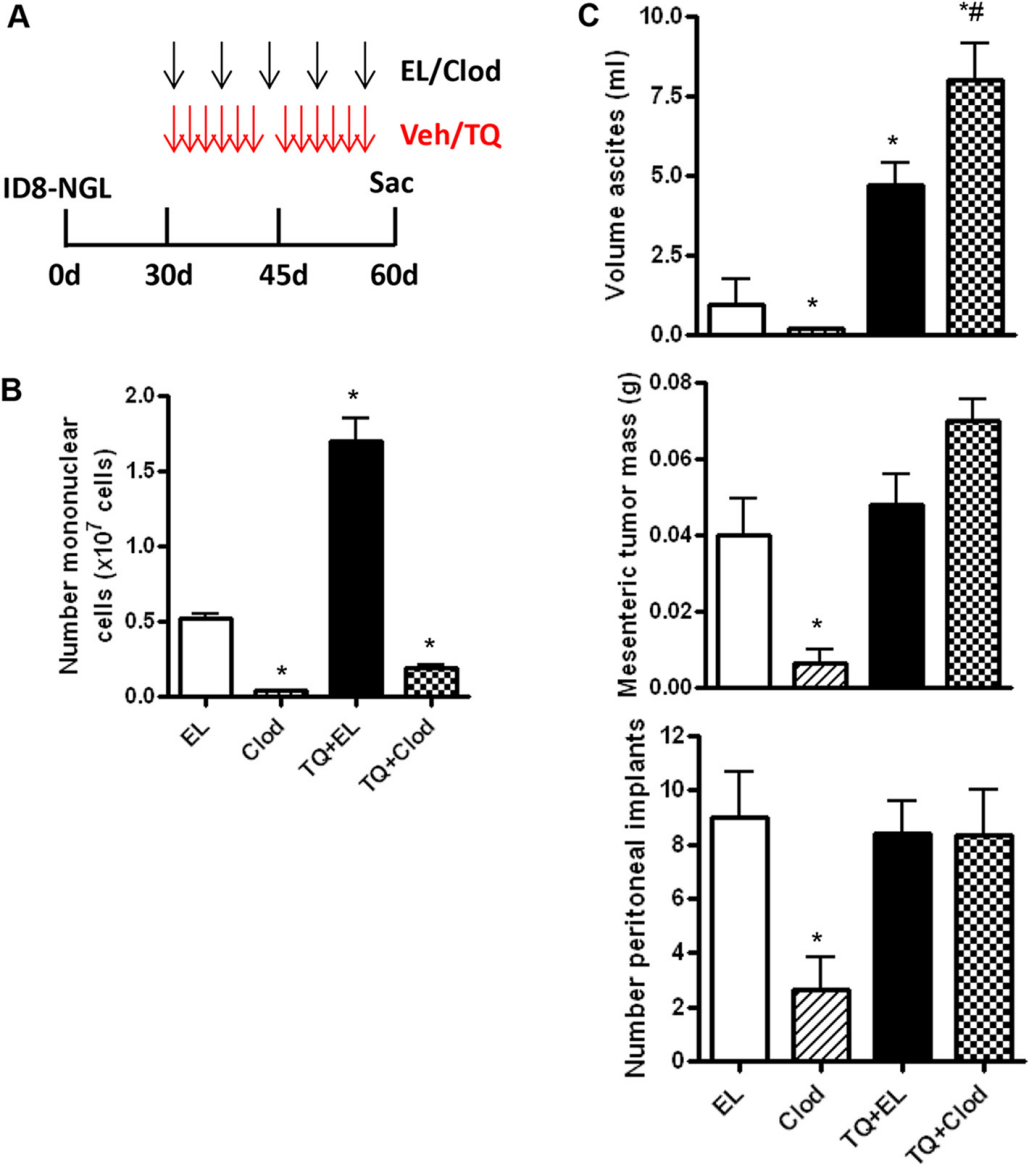
Effects of macrophage depletion on TQ-induction of ascites. **a** Schematic of weekly clodronate/empty liposome (EL) and thrice weekly vehicle or 20 mg/kg TQ treatment for 30 days. **b** Clodronate depletes mononuclear cells in ascites collected from mice treated weekly with empty liposomes (EL), liposomal-conjugated clodronate (Clod), thrice weekly with TQ (20 mg/kg) and empty liposomes and TQ and clodronate for 30 days, as quantified in differential cell counts of H&E-stained cytospin slides. **c** Volume of ascites, number of peritoneal implants and mesenteric tumor mass in mice treated with clodronate/empty liposome (EL) and thrice weekly vehicle or 20 mg/kg TQ treatment for 30 days. *p < 0.01 compared to vehicle; #*p* < 0.01 compared to TQ and empty liposome treatment alone, both Mann–Whitney test

Our results indicate that TQ-stimulation of ascites formation was accompanied by changes in peritoneal macrophage populations permissive for tumor progression. In order to directly examine the role of macrophages on the effects of TQ, we utilized a macrophage depletion strategy with liposomal clodronate [24]. Clodronate was administered IP from 30–60 days following injection of ID8-NGL cells (Fig. 7a). The following 4 treatment groups were used: empty liposomes (EL; liposomes and PBS), EL + TQ, CLOD (liposomes and clodronate), and CLOD + TQ. First, we confirmed that CLOD markedly reduced overall numbers of mononuclear cells in ascites fluid cytospin slides, in both the CLOD and CLOD + TQ groups (Fig. 7b). CLOD alone significantly inhibited ascites formation, mesenteric tumor mass, and the number of peritoneal implants (Fig. 7b), indicating the important role of macrophages in ovarian cancer progression. Despite this, the combination of TQ and CLOD significantly augmented ascites formation above that of EL + TQ, while no differences in mesenteric tumor mass and peritoneal implants were observed (Fig. 7b).

**Fig. 8:**
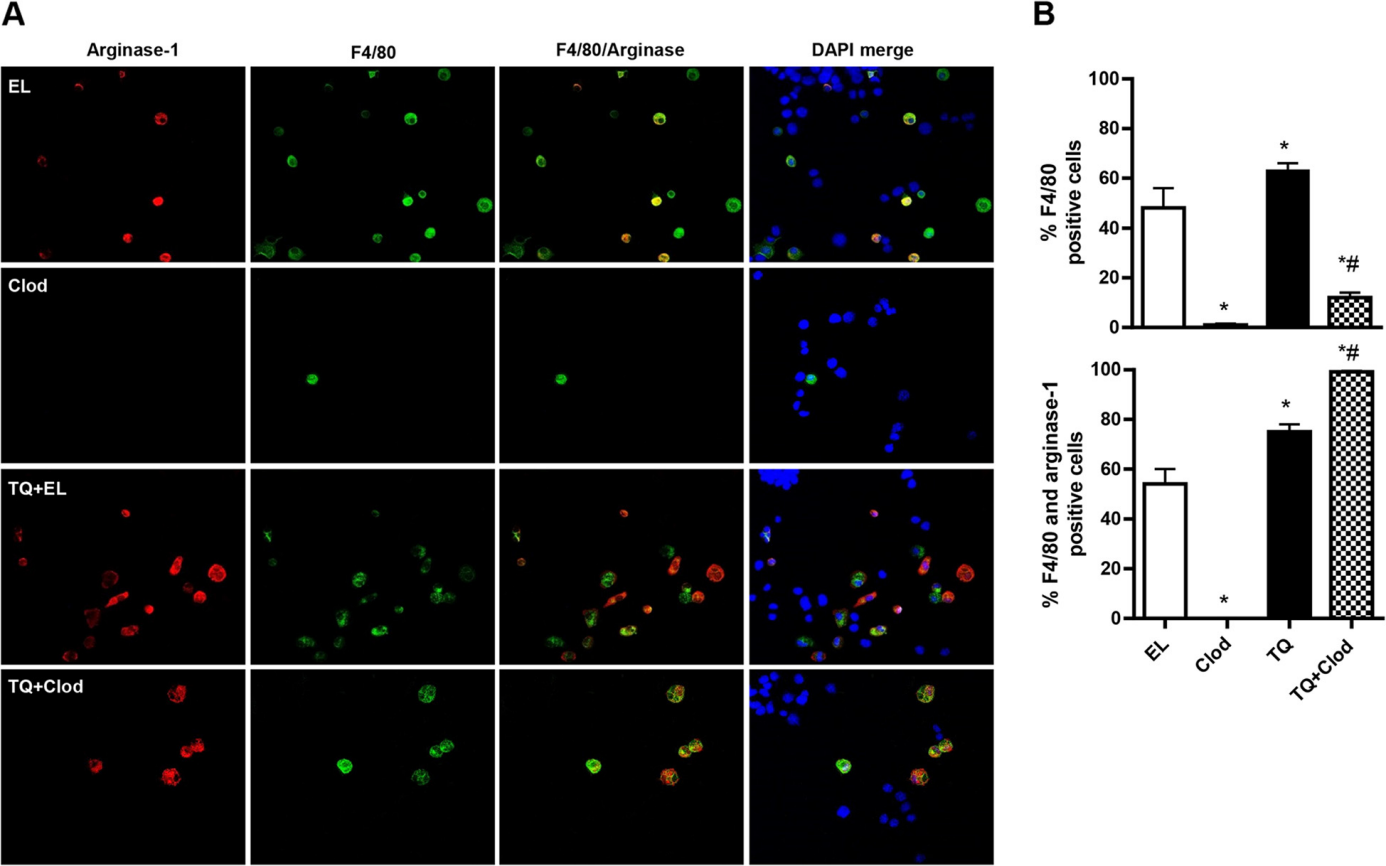
Clodronate preferentially depletes non-M2 macrophages in mice co-treated with TQ. **a** IF analysis of expression of the macrophage markers F4/80 (green) and arginase-1 (red) in ascites harvested from mice treated with weekly clodronate/empty liposome (EL) and thrice weekly vehicle or 20 mg/kg TQ treatment for 30 days. Ascites contains of mixture of F4/80-positive macrophages and F4/80-negative cells (for example, tumor cells). DAPI-stained nuclei are in blue. **b** Quantification of the percentage of cells staining positive for F4/80, and for both F4/80 and arginase-1. Values were determined from 5 representative fields at high power (×40). *p < 0.01 relative to vehicle; all *p* values determined by Mann–Whitney test

We then examined ascites fluid for macrophage phenotype in the 4 treatment groups. As shown in Fig. 8a and b, CLOD significantly reduced the percentage of F4/80-positive cells observed. However, when the remaining F4/80-positive cells were examined for co-expression of the M2 marker, arginase-1, we found that the macrophages that were present in the CLOD + TQ were almost 100 % arginase-1-positive. In contrast, in the CLOD alone group, no macrophages stained positive for arginase-1. These data are further evidence that TQ can alter the composition of the tumor microenvironment to be permissive for tumor progression.

### TQ increases pro-tumorigenic inflammatory mediators in ascites fluid

**Fig. 9:**
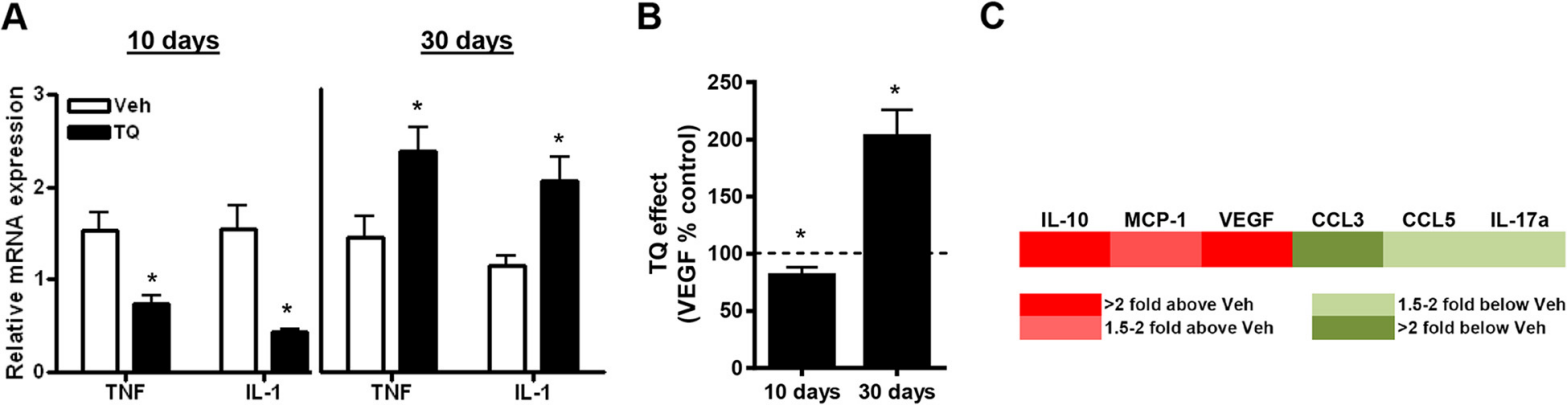
Increased pro-tumorigenic inflammatory mediators in long-term TQ-treated mice. **a** QPCR measurement of mRNA levels of the established NF-κB target genes, IL-1β and TNF-α, in harvested peritoneal macrophages from mice treated with thrice weekly 20 mg/kg TQ or vehicle (Veh) for 10 or 30 days. Values were expressed relative to corresponding GAPDH levels. **b** VEGF levels in ascites fluid was measured by ELISA and expressed relative to corresponding protein levels. Values are mean + SE for 3–5 mice per group. *p < 0.01 relative to vehicle; all *p* values determined by Mann–Whitney test. **c** Cytokine/growth factor profiles in ascites from vehicle or TQ treated mice (30 days)

We next compared the inflammatory profiles of ascites fluid between mice with TQ for 10 or 30 days. As shown in Fig. 9a, QPCR analysis of steady-state mRNA levels of the established NF-κB targets, TNF-α and IL-1β, in isolated macrophages revealed that 10 day TQ treatment led to reduced expression of TNF-α and IL-1β. We also examined expression of another classical pro-tumorigenic NF-κB target gene, VEGF, implicated in angiogenesis and increasing vascular permeability [25], in ascites fluid. VEGF levels were modestly, but significantly, reduced in ascites fluid after 10 days (Fig. 9b). In contrast, 30 days TQ treatment led to overall increases in expression of TNF-α and IL-1β in macrophages and VEGF in ascites fluid (Fig. 9a&b). This observation of elevated NF-κB targets with 30 day TQ treatment was consistent with increased NF-κB reporter activity in isolated macrophages harvested from the ascites fluid ID8-injected NGL reporter mice (Additional file 1: Figure S1E).

We also analyzed the cytokine profile of ascites fluid harvested from vehicle and 30 day TQ-treated mice using a commercially available cytokine array plate. As shown in Fig. 9c, there was evidence of coordinated drug-induced regulation of cytokine expression. Ascites from mice treated with TQ showed increased levels of VEGF and known pro-tumorigenic cytokines, IL-10 and MCP-1 [26–28] . Furthermore, levels of several anti-tumor cytokines, such as CCL3, CCL5 and IL-17α were reduced by TQ treatment. Overall levels of multiple other cytokines/growth factors also measured on the array plate, such as IL-2, IL-4, IL-6, IFN-γ, EGF, SCF, leptin, PDGF-BB, resistin and β-NGF, were not altered by drug treatment.

## Discussion and conclusions

TQ is a promising anti-cancer drug known to have multiple anti-tumorigenic effects in cancer cells, including inhibition of NF-κB [4–9]. We have previously shown that activity of NF-κB in tumors is increased during cancer progression in the ID8-NGL syngeneic mouse model of ovarian cancer [10]. In order to identify new drug combinations to treat ovarian cancer, we recently reported the ability of TQ to synergize with cisplatin and limit tumor progression following 30 days treatment [15]. However, we also demonstrated an unexpected increase in ascites accumulation and tumor NF-κB activity in response to TQ alone.

The present study was designed to investigate mechanisms by which TQ induces these paradoxical stimulatory effects on ascites production and NF-κB activity *in vivo*. We compared the effects of short-term (10 day) and prolonged (30 day) treatment since we have previously observed the expected anti-tumor effects such as NF-κB inhibition in tumors and decreased expression of M2-like pro-tumor peritoneal macrophage markers in 10 day TQ-treated mice [10]. Our experiments strongly suggested that direct tumor effects of TQ were not a mechanism for increased ascites and NF-κB activity at 30 days treatment. First, TQ induced similar effects on growth inhibition and apoptosis induction at both time points. Second, there was persistence of TQ-mediated inhibition of NF-κB reporter activity for at least three weeks in cultured ID8-NGL cells.

Instead of direct tumor effects, multiple lines of evidence indicated that prolonged TQ treatment induced pro-tumorigenic changes in the tumor microenvironment. Increased ascites formation was accompanied by elevated NF-κB activity in peritoneal macrophages, increased expression of M2-like macrophage markers and levels of pro-tumorigenic soluble factors such as VEGF, IL-10 and MCP-1 in ascites fluid [25, 28, 29], and increased infiltration of pro-tumor M2 macrophages into tumors. This sharply contrasted with reduced expression of M2 macrophage markers in 10 day TQ-treated mice. Our observations are suggestive of an elevated inflammatory response with prolonged exposure to TQ, and are in agreement with studies demonstrating a key role for immune cell infiltration in progression of ovarian tumors [20–22]. Moreover, our observations are consistent with a recent study in a murine lung cancer model showing that while short-term exposure to the NF-κB inhibitor bortezomib produces the expected inhibition of tumor cell growth, prolonged treatment results in pro-inflammatory effects and promotes tumor progression [30]. Despite the similarities in these findings, it remains a formal possibility that these deleterious side-effects of TQ and bortezomib may not be related to NF-κB inhibition, since they have multiple off-target effects [11–13, 31]. More investigation into these mechanisms is clearly warranted to inform design of the safest and most efficacious ongoing therapeutic strategies.

We anticipated that depletion of macrophages using clodronate might abrogate the increased ascites accumulation. However, while depletion of macrophages by clodronate produced the expected beneficial effects on tumorigenesis [24], we found that ascites was not inhibited, but was rather enhanced by combined clodronate and TQ treatment. One possibility is that the macrophages were not essential at that stage for the observed phenotype. However, while clodronate can effectively temporarily deplete resident macrophages, naïve macrophages are called in to replace those that have been lost. It is our belief that the clodronate treatment depleted both pro- and anti-tumor macrophage populations, and that naïve macrophages repopulating the peritoneal cavity in the TQ-treated mice were strongly preferentially shifted toward the M2 phenotype. Therefore although the absolute numbers of these macrophages were smaller than in an untreated animal, the higher proportion of M2-like macrophages generates an overall pro-tumorigenic microenvironment.

Our data suggest that microenvironmental stimuli promoted a sub-population of proliferating tumor cells with increased NF-κB activity that limited overall tumor response to TQ. Upregulation of NF-κB activity is a well-established mechanism of drug resistance [23]. The development of drug resistance may explain the apparently contradictory observation of reduced tumor proliferation and increased apoptosis following 30 days TQ treatment, but no significant changes in tumor mass and number of peritoneal tumor implants compared to vehicle at this endpoint. We also acknowledge the limitation of measuring tumor burden by gross dissection, with a bias towards more established tumors being harvested and/or counted such that developing micro-tumors would not be detected. The fact that experimental mice were sacrificed immediately following the 30 days of drug treatment, due to the large ascites burden of TQ-treated mice, precluded the possibility of detecting potential development of multiple smaller tumors.

We believe that increased ascites following prolonged TQ treatment was a result of increased VEGF production by M2-like macrophages in association with peritoneal tumor cells and induced by local, peritoneal effects, rather than by systemic toxic side-effects of TQ. While TQ enters the circulation following intra-peritoneal injection [32], and TQ is known to have anti-inflammatory and immune modulatory effects [33], this is the first description of ascites formation in any immunodeficient or immunocompetent mouse preclinical tumor model of TQ treatment. Furthermore, other studies have shown no toxicity following similar intraperitoneal dosing of TQ [32, 34], and we detected no signs of TQ-induced toxicity either in the whole animal or on gross or histological examination of various organs at sacrifice.

**Fig. 10:**
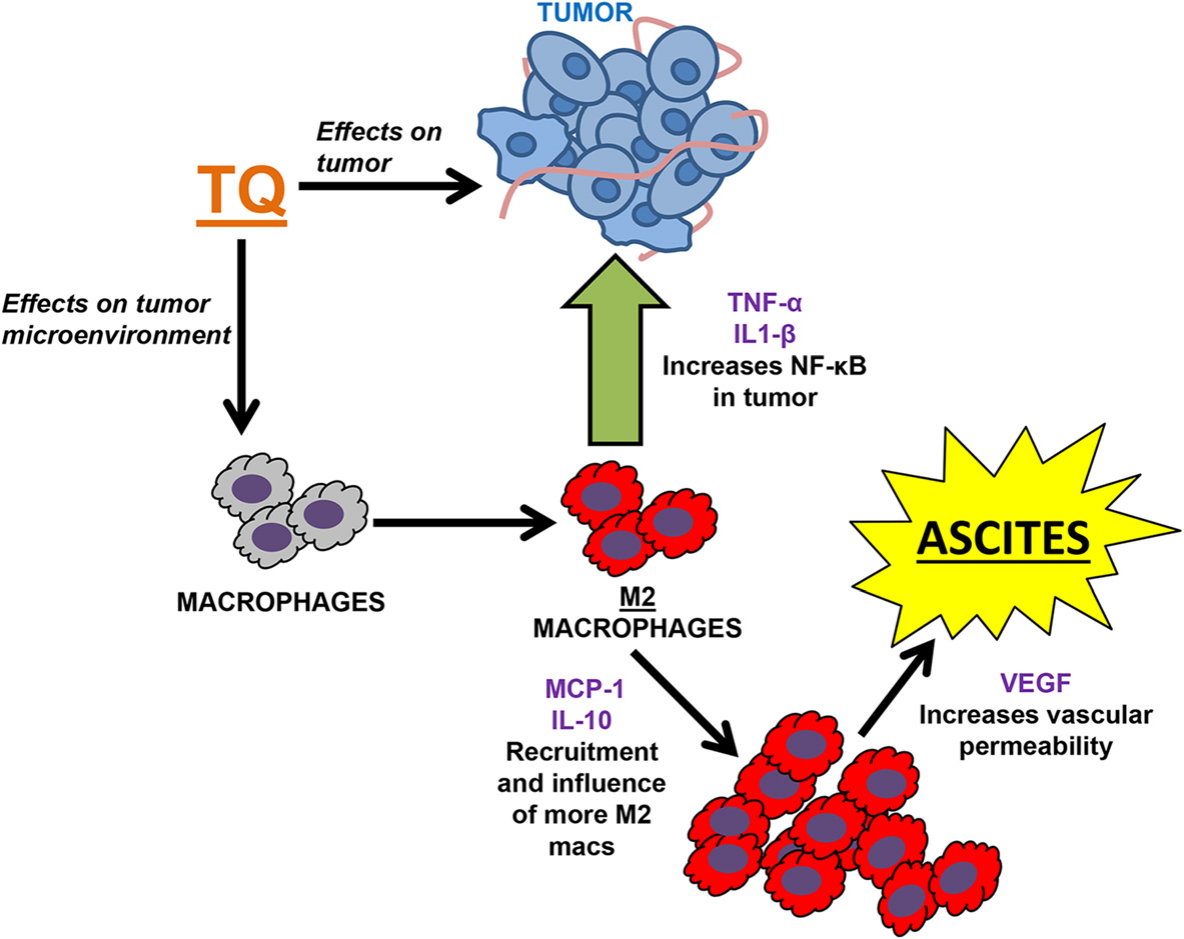
The contribution of peritoneal macrophages to the deleterious, pro-tumor effects of 30 day TQ treatment. Extended TQ treatment induced a substantial shift in macrophage phenotype within the tumor microenvironment, to pro-tumor M2-like macrophages. M2 macrophages showed increased levels of macrophage recruiting factors that creates a positive feedback loop further increasing macrophage numbers, and of VEGF, an important angiogenic factor that induces leaky vasculature characteristic of ascites. M2-like macrophages also produced increased signals such as TNFα, which have the ability to increase NF-κB activity in tumors, leading to a treatment-resistant subpopulation of tumor cells

Based on our results, we have developed the following conceptual framework to explain deleterious side-effects following TQ treatment in ovarian cancer (Fig. 10). Our findings indicate that the phenotype of macrophages within the tumor microenvironment was significantly changed as a result of extended TQ treatment, leading to a significantly greater proportion of pro-tumor M2-like cells. These cells then produced increased NF-κB activating signals such as TNF-α, which have direct effects on the tumor cells and overwhelm the inhibitory functions of TQ on the epithelial cells themselves. It is also possible that upon TQ treatment, there is selection for tumor cells with elevated NF-κB activity that are intrinsically insensitive to inhibition by TQ, thus developing a drug resistant population.

In another clinically relevant outcome, these M2-like macrophages have increased expression of VEGF which is an important angiogenic factor known to induce leaky vasculature and thus represents a mechanism by which the increased ascites formation occurs. Finally, they also increase levels of macrophage recruiting factors such as MCP-1 and IL-10 creating a positive feedback loop in which increased numbers of pro-tumor macrophages are recruited.

Overall, our data support the conclusion that adverse microenvironmental effects following sustained systemic NF-κB inhibition arising in macrophages can significantly limit the efficacy of this therapeutic strategy in ovarian cancer. A novel, dual intervention strategy enabling inhibition of NF-κB in tumor epithelium while coincidently targeting macrophage functions could prove effective. Towards development of such an approach, our group has shown that the context-dependent targeting of NF-κB in macrophages induces significant anti-tumor activity in transgenic mouse models [35, 36], and have characterized highly promising, novel mannosylated polymer nanoparticles (MnNP) to specifically deliver siRNA to tumor-associated macrophages *in vitro* and *in vivo* [37]. Because of the considerable interest in using systemic NF-κB inhibitors as mono-therapy or in combination with other chemotherapeutic drugs in clinical trials in ovarian cancer patients [16, 38], the results presented in this current manuscript are highly relevant to the clinic.

## Methods

### Cell culture

Mouse ovarian cancer cells stably expressing a NF-κB reporter plasmid, ID8-NGL [10], were cultured in 10 % FBS-supplemented DMEM High-Glucose medium with 400 μg/ml G418, and passaged by standard techniques. Cultured ID8-NGL cells were treated with increasing concentrations of the NF-κB inhibitor, thymoquinone (TQ; Sigma Chemical Co., Cat# 274666).

### Animal model and drug treatment

Wild-type C57BL/6 mice were injected intra-peritoneally (IP) with 1×10^7^ ID8-NGL cells suspended in 200 μl sterile PBS [10]. 30 days after ID8-NGL injection, mice were randomized into the following treatment groups: 10 day vehicle (PBS thrice weekly IP for 10 days), 30 day vehicle (PBS thrice weekly IP for 10 days), 10 day TQ (20 mg/kg TQ thrice weekly IP for 10 days), and 30 day TQ (20 mg/kg TQ thrice weekly IP for 30 days). No signs of toxicity were observed in the drug-treated mice at either duration of TQ exposure. In separate experiments, 30 days after ID8-NGL injection, mice were randomized and treated as follows for 30 days: sterile PBS-containing liposome (empty liposome, EL, weekly IP), 30 day TQ + EL (20 mg/kg TQ thrice weekly and EL weekly IP), clodronate (dichloromethylene diphosphonic acid; Sigma Chemical Co., Cat#D4434)-containing liposomes (CLOD weekly IP) and 30 day TQ + CLOD (20 mg/kg TQ thrice weekly and CLOD weekly IP). Empty and clodronate-liposomes were prepared as previously described [24]. Furthermore, confirmatory experiments were performed in a complementary model where C57BL/6 NGL reporter mice [39] were injected intra-peritoneally (IP) with 1×10^7^ wild-type ID8 cells, and as treated for 30 days with TQ as above.

Tumor progression was monitored by body weight and abdominal girth measurements. At time of sacrifice, abdominal ascites fluid was extracted with hypodermic syringe, and volume measured. If no measurable ascites was present, peritoneal lavages were performed by injecting 8 ml PBS intra-peritoneally and carefully extracting the fluid with a hypodermic syringe [10]. Tumor implants in the peritoneal wall and the mesentery were harvested and snap frozen or formalin-fixed for further analysis. To determine possible toxicity due to TQ treatment, we monitored behavior and body condition score of the mice, along with gross and histological examination of the liver, heart, lungs and small and large intestines. The experimental protocol was reviewed and approved by the Institutional Animal Care and Use Committee at Vanderbilt University (IACUC #M/10/395).

### Luciferase assays

Luciferase activity was measured in harvested tumors following tissue homogenization in 1 ml reporter lysis buffer, and in whole cell protein extracts from cultured ID8-NGL cells treated with increasing concentrations of TQ, using the Promega Luciferase Assay system (Cat#4030). Activity was analyzed using a GloMax Luminometer (Promega, Madison, WI). Results were expressed as relative light units (RLU) normalized for protein content, as measured by the Bradford assay (Bio-Rad, Cat# 500–0002).

### Analysis of ascites/peritoneal lavage fluid

Ascites or peritoneal lavage fluid was centrifuged at 1500 rpm for 5 min to separate cells from supernatant. Where applicable, red blood cells were lysed by ACK lysing buffer according to manufacturer’s instructions (Life Technologies, Cat# A10492-01). An aliquot of cells were suspended in PBS with 1 % BSA (Sigma Chemical Co., Cat# 05470) for total cell counts using a grid hemocytometer. Cells were then either snap-frozen for RNA extraction, or centrifuged onto microscope slides using a Thermo Cytospin II Cytocentrifuge (500 rpm for 10 min) for differential counts of inflammatory cells in hematoxylin and eosin-stained cells or immuofluorescence analysis. Cytokine/growth factor composition in the soluble fraction of ascites harvested from drug-treated mice was analyzed by mouse cytokine array (Cat# EA-4003) and VEGF ELISA (Cat# EA-2401) plates (Signosis Inc.). For each sample, levels of cytokines were normalized to corresponding total protein levels measured by Bradford protein assay.

### RNA extraction and quantitative RT-PCR (QPCR)

RNA from snap-frozen tumors, ascites fluid or peritoneal lavages was isolated using the RNeasy Mini kit (Qiagen, Valencia, CA) and QPCR performed as described the comparative 2^ΔΔCt^ method [40]. Steady-state mRNA levels of the M1 macrophage marker, CC chemokine ligand 3 (CCL3), the M2 macrophage markers, mannose receptor (mann-R) and interleukin-10 (IL-10), and the established NF-κB targets, TNF-α and IL-1β, were expressed relative to corresponding GAPDH levels the comparative 2^ΔΔCt^ method [40]. Relative expression values were also normalized to levels of the epithelial marker cytokeratin-18 (CK18) to account for the epithelial (tumor) cell component of ascites or peritoneal lavage fluid. Primer sequences used were as previously described [10, 39, 41].

### Immunofluorescence analysis

Processing, embedding and sectioning of formalin-fixed ID8-NGL tumor tissue, and hematoxylin and eosin staining for histology, were performed in The Allergy/Pulmonary & Critical Care Med Division Immunohistochemistry Core at Vanderbilt [42]. Immunofluorescence analysis of formalin-fixed paraffin-embedded tumor tissue or in cytospin slides of ascites fluid or isolated macrophages was performed using standard techniques [10, 43]. The following primary antibodies were used: rabbit polyclonal anti-Ki67/Mib-1 (Abcam, Cat# ab16667; 1:200 dilution), rabbit polyclonal anti-cleaved caspase-3 (Cell Signaling Technology, Cat# 9661; 1:100 dilution), rat polyclonal anti-F4/80 (AbD Serotec, Cat# MCA497, 1:200), and rabbit polyclonal anti-arginase-1 (Santa Cruz, Cat# sc-20150; 1:100 dilution). Secondary antibodies used were goat anti-rat Alexa Fluor 488 (Life Technologies, Cat# A-11006), goat anti-mouse Alexa Fluor 594 (Life Technologies, Cat# 11020), and goat anti-rabbit Alexa Fluor 488 (Life Technologies, Cat# 11070) (all 1:200 dilution). Images were acquired and analyzed as previously described [10, 43]. For quantifying the percentage of, where applicable, tumor cells or macrophages positive for these proteins, at least 5 independent fields were assessed with at least 200 cells counted per sample.

### Western blotting

In ID8-NGL cells treated with TQ (50 μM) in vitro, or ID8-NGL tumors, whole cell protein isolation, subcellular fractionation, western blotting and signal detection were performed as described [44, 45]. Primary antibodies used were rabbit polyclonal anti-PARP (Cell Signaling Technology; Cat# 9542; 1:1000 dilution), and mouse monoclonal anti-β-actin (Sigma Chemical Co., Cat# A5441 1:10000 dilution) as loading control.

### Cell viability assays

Sulforhodamine B (SRB) assays were used to determine cell viability in cultured ID8-NGL cells treated with increasing concentrations of TQ, as previously described [46].

### Statistical analysis

Unless otherwise indicated, values shown for *in vitro* experiments were the mean + SE of 3 independent experiments, with comparison of groups performed by 2-tailed Student’s *t* test. Comparison of groups in *in vivo* experiments was performed by 2-tailed Mann–Whitney test. A *p* value < 0.05 was considered statistically significant.

## Additional file

Additional file 1:
**Effect of 30 day treatment with 40 mg/kg TQ or vehicle in NGL reporter mice injected with ID8 cells.** (A) Quantification of ascites fluid volume at sacrifice showed increased ascites with TQ treatment, but no significant differences in (B) the number of peritoneal implants or (C) mesenteric tumor mass. (D) QPCR analysis of the mRNA expression of the markers of M2 macrophages, mannose-receptor (mann-R) and interleukin-10 (IL-10) and M1 macrophages (CCL3) in RNA extracted from peritoneal lavages or ascites fluid. Values were normalized to corresponding levels of GAPDH mRNA expression. (E) Luciferase activity of the NF-κB reporter was measured in isolated macrophages from ascites or peritoneal lavage fluid, and expressed relative to cellular protein. Values are mean+SD for 5 mice per group. **p* < 0.01 relative to vehicle-treated mice; NS: not significant relative to vehicle, Mann-Whitney test. (PDF 117 kb)

## Abbreviations

IL: interleukin
NF-κB: nuclear factor-kappaB
NGL: NF-κB-GFP-Luciferase
TQ: thymoquinone
VEGF: vascular endothelial growth factor
