# Supplementary material for: Microenvironmental effects limit efficacy of thymoquinone treatment in a mouse model of ovarian cancer
Source: Mol Cancer. 2015 Nov 9;14:192. doi: 10.1186/s12943-015-0463-5 (PMC4640396; doi:10.1186/s12943-015-0463-5)
Supplement: Additional file 1: — Effect of 30 day treatment with 40 mg/kg TQ or vehicle in NGL reporter mice injected with ID8 cells. (A) Quantification of ascites fluid volume at sacrifice showed increased ascites with TQ treatment, but no significant differences in (B) the number of peritoneal implants or (C) mesenteric tumor mass. (D) QPCR analysis of the mRNA expression of the markers of M2 macrophages, mannose-receptor (mann-R) and interleukin-10 (IL-10) and M1 macrophages (CCL3) in RNA extracted from peritoneal lavages or ascites fluid. Values were normalized to corresponding levels of GAPDH mRNA expression. (E) Luciferase activity of the NF-κB reporter was measured in isolated macrophages from ascites or peritoneal lavage fluid, and expressed relative to cellular protein. Values are mean+SD for 5 mice per group. *p < 0.01 relative to vehicle-treated mice; NS: not significant relative to vehicle, Mann-Whitney test. (PDF 117 kb) [file 12943_2015_463_MOESM1_ESM.pdf]

Supplementary Figure 1

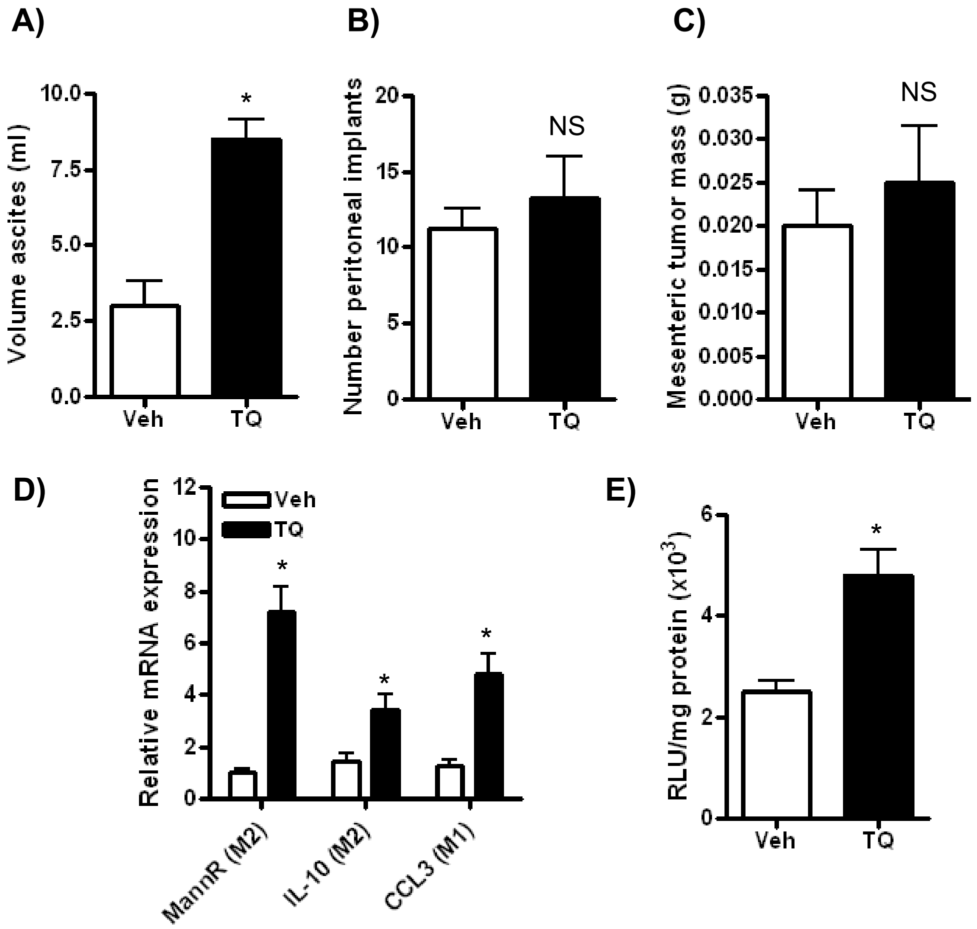

Effect of 30 day treatment with 40 mg/kg TQ or vehicle in NGL reporter mice injected with ID8 cells. (A) Quantification of ascites fluid volume at sacrifice showed increased ascites with TQ treatment, but no significant differences in (B) the number of peritoneal implants or (C) mesenteric tumor mass. (D) QPCR analysis of the mRNA expression of the markers of M2 macrophages, mannose-receptor (mann-R) and interleukin-10 (IL-10) and M1 macrophages (CCL3) in RNA extracted from peritoneal lavages or ascites fluid. Values were normalized to corresponding levels of GAPDH mRNA expression. (E) Luciferase activity of the NF-κB reporter was measured in isolated macrophages from ascites or peritoneal lavage fluid, and expressed relative to cellular protein. Values are mean+SD for 5 mice per group. * $p < 0.01$ relative to vehicle-treated mice; NS: not significant relative to vehicle, Mann-Whitney test.
